# CodingMotif: exact determination of overrepresented nucleotide motifs in coding sequences

**DOI:** 10.1186/1471-2105-13-32

**Published:** 2012-02-14

**Authors:** Yang Ding, William A Lorenz, Jeffrey H Chuang

**Affiliations:** 1 Department of Biology, University of Pennsylvania, Philadelphia, PA 19104, USA; 2 Department of Mathematics and Computer Science, Denison University, Granville, OH 43023, USA; 3 Department of Biology, Boston College, Chestnut Hill, MA 02467, USA

## Abstract

**Background:**

It has been increasingly appreciated that coding sequences harbor regulatory sequence motifs in addition to encoding for protein. These sequence motifs are expected to be overrepresented in nucleotide sequences bound by a common protein or small RNA. However, detecting overrepresented motifs has been difficult because of interference by constraints at the protein level. Sampling-based approaches to solve this problem based on codon-shuffling have been limited to exploring only an infinitesimal fraction of the sequence space and by their use of parametric approximations.

**Results:**

We present a novel *O*(*N*(log *N*)^2^)-time algorithm, CodingMotif, to identify nucleotide-level motifs of unusual copy number in protein-coding regions. Using a new dynamic programming algorithm we are able to exhaustively calculate the distribution of the number of occurrences of a motif over all possible coding sequences that encode the same amino acid sequence, given a background model for codon usage and dinucleotide biases. Our method takes advantage of the sparseness of loci where a given motif can occur, greatly speeding up the required convolution calculations. Knowledge of the distribution allows one to assess the exact non-parametric p-value of whether a given motif is over- or under- represented. We demonstrate that our method identifies known functional motifs more accurately than sampling and parametric-based approaches in a variety of coding datasets of various size, including ChIP-seq data for the transcription factors NRSF and GABP.

**Conclusions:**

CodingMotif provides a theoretically and empirically-demonstrated advance for the detection of motifs overrepresented in coding sequences. We expect CodingMotif to be useful for identifying motifs in functional genomic datasets such as DNA-protein binding, RNA-protein binding, or microRNA-RNA binding within coding regions. A software implementation is available at http://bioinformatics.bc.edu/chuanglab/codingmotif.tar

## Background

Coding sequences have been shown to harbor numerous regulatory sites in their nucleotide sequences for functions such as RNA localization [1], translation efficacy [2], mRNA splicing [3], mRNA stability [4], and accessibility to the translation machinery [5]. The existence of such regulatory sites suggests that searching for cis-regulatory elements only in promoter regions or UTRs overlooks a great deal of important biology. This regulatory importance within coding regions is perhaps not surprising, as coding sequences are comparable in length to both UTRs and promoter regions. Although there is substantial variation in the length of UTRs and coding sequences, on average human coding sequences are ~ 1000 bp long, while 3' UTRs are ~ 800 bp and 5' UTRs are ~ 100-200 bp. In 56% of transcripts the coding region is longer than the 5' and 3' UTRs combined (Ensembl v63).

High-throughput studies of both RNA and DNA have also shown evidence of functional sites in coding regions, indicating the need for computational methods to identify such sites. Some of the RNA studies include those showing binding of proteins or microRNAs to mRNA coding regions [6,7]. At the DNA level, transcription factor binding site mapping has shown that coding regions contain extensive binding sites in bacteria [8], DNAse hypersensitivity measurements have shown that the transcription factors are likely to bind into the first several hundred bases of human coding regions [9], and transcription factor chromatin immunoprecipitation studies have shown similar behavior in Drosophila coding regions [10]. It has also been shown that the sequence motifs overlapping the stop codon may influence protein yield [11].

Identifying functional motifs in coding sequences computationally has been challenging due to the lack of appropriate algorithms to separate nucleotide-level signals from those caused by the amino acid sequences. Here we use the term motif to refer to a short possibly degenerate sequence element that may or may not be functional. Sequence conservation approaches that calibrate for the amino acids are one promising technique for identifying functional motifs, as it was shown that conservation can detect exonic splicing, microRNA binding, and DNA replication-associated motifs [12-14]. However, the function of a motif identified by sequence conservation is often non-obvious. This is in contrast to motif identification by overrepresentation, for which functions can be assigned based on the manner in which the sequence set was identified. For example, sequences captured in RNA immunoprecipitation or chromatin immunoprecipitation are likely to contain overrepresented motifs relevant to the specific proteins binding to the RNA or DNA. Development of a motif overrepresentation algorithm for coding regions would therefore be of considerable value.

A few groups [15-17] have attempted to separate the amino acid and nucleotide-level pressures on motif copy number, using codon usage biases as a starting point. However all of these methods have been based on sampling sequences whose codons have been shuffled while preserving the amino acid sequence. Such an approach is limited by the number of shuffled sequences that can be sampled in a feasible amount of time. Proteins are on average more than 300 codons long [18] and almost all codons are at least 2-fold degenerate, yielding exponentially many possible codon sequences per protein. Prior studies have sampled only an infinitesimal portion of the sequence space, e.g. Down et al compared real sequences to a single shuffle [15], Itzkovitz et al compared to 20 shuffles [17], and Robins et al compared to 50 shuffles [19]. To compensate for the limited sampling depth, parametric approximations were used for the motif count null distribution based on the behavior of the sampled sequences [17,19]. However, the adequacy of such parametric approximations is unclear, as they depend on many factors, including the number of samples, the length of the original sequence, the encoded amino acid sequence, the shape of the distribution implied by the parametric approximation, and the true prevalence of the motif to be tested. The validity of the parametric approximation as well as the required depth of sampling are in general unknown for a given dataset and motif.

Methods based on comparisons to empirical motif counts in control exonic (and sometimes intronic) sequences have also been developed [3,20], though such approaches require additional functional knowledge of the control sequences and, like the shuffling approaches, are limited by size of the control set.

Algorithms that simply ignore the amino-acid sequence have been applied to coding sequences as well. For example Jambhekar et al [21] used MEME, which is designed for noncoding sequences [22], to search for RNA localization zipcodes. However they concluded that MEME, even when combined with RNA folding simulations, was unreliable for this purpose [1].

In this work we present a novel enumerative method, CodingMotif, to detect functional noncoding motifs in coding sequences, solving the problems associated with sampling approaches. The algorithm exactly calculates the distribution of a motif's occurrence frequency over all coding sequences that code for the amino acid sequence, given a null model of codon usage. This approach allows for exact evaluation of the overrepresentation or underrepresentation p-value for a motif in any length of sequence *N*. This removes the need for sampling or for parametric approximations, providing a key advance over prior approaches. Our algorithm is able to efficiently calculate the distribution in *O*(*N*(log *N*)^2^) time through a novel dynamic programming algorithm. We describe how to speed up the calculation by taking advantage of motif sparseness as well. Importantly, the program also takes into account dinucleotide biases, which are built into the model through a codon-to-codon Markov process. We show that CodingMotif assesses motifs more accurately than sampling approaches in both eukaryotic and prokaryotic datasets.

## Results and discussion

### Independent codon model

**Figure 1:**
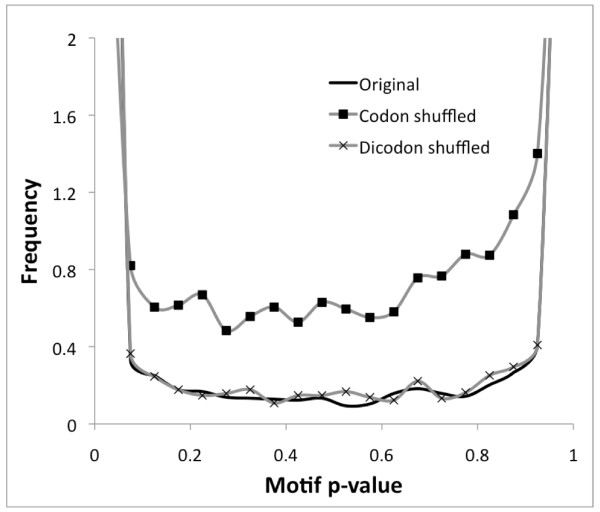
**Distribution of motif overrepresentation p-values for mouse chr19 coding sequence with the Independent Codon Model null**. Three ICM p-value distributions are shown: the p-values for the original coding sequences; the p-values after shuffling synonymous codons across coding sequences; and the p-values after shuffling dicodons across coding sequences.

As a first approach to the problem, we developed a motif overrepresentation algorithm based on an Independent Codon Model (ICM), in which our null assumption was that codons do not influence the codons at adjacent positions (see Methods). To determine the effectiveness of this assumption, we first analyzed k-mer strings ((A,G,C,T)^*k *^) for overrepresentation in the coding sequences of mouse chromosome 19 (623,203 codons; 1331 coding sequences). Prior studies have focused on analyzing overrepresentation for k-mers as well [17,19].

k-mer scores exhibited a strong bimodal behavior under the ICM null, with the vast majority of p-values close to either 0 or 1. The distribution of p-values for these k-mers is shown in Figure 1. and labeled as "original sequence." For example, 32% of 6-mers have p-value < 0.05, and 19% of 6-mers have p-value > 0.95. The large number of motifs with strong copy number biases suggests that the ICM model may not be an adequate null model for detecting motifs under selection. To clarify the reason for the bimodal behavior, we shuffled the codons while keeping the amino acid sequences fixed, yielding a "codon shuffled" sequence. When the overrepresentation algorithm was run on this shuffled sequence, the bimodality of the scores was substantially decreased (Figure 1). This suggests that there are systematic dinucleotide biases at the boundaries of neighboring codons that significantly impact the p-values calculated for the original sequence. Such biases may include selective pressures on motifs, which is what we seek to identify; however the large number of motifs with scores altered by the codon shuffling suggests that there are neutral effects as well.

We hypothesized that the ICM null model may be inadequate for detecting motifs under selection because it ignores neutral dinucleotide mutation biases. To clarify the effect of dinucleotide biases, we shuffled the original coding sequences while maintaining dicodon frequencies (and consequently dinucleotide frequencies; see Methods) using the method of [23]. When the overrepresentation program was applied to this dicodon-shuffled sequence, the distribution of motif p-values was very close to that for the original sequence (Figure 1). Thus dinucleotide biases at codon boundaries are responsible for much of the behavior of motif p-values for the original sequence. This finding is consistent with previous works showing that the CpG effect has a strong influence on motif occurrence in coding regions [12], as well as earlier studies that have analyzed dicodon correlations in coding regions [24-26]. Therefore when detecting motifs whose copy numbers are increased due to selective pressures, it is important to include dinucleotide effects in the null model; otherwise many of the motifs inferred to be under selection would be false positives.

### Dinucleotide-corrected codon model

To handle this problem, we developed a method to calculate the motif frequency distribution that would be generated by a null model that includes dinucleotide biases. The algorithm uses as its null a Markov model that closely preserves the expected codon usage and dinucleotide frequencies in the reference sequence. We refer to this as the dinucleotide-corrected codon model (DCM). Full details of the DCM are given in the Methods.

**Figure 2:**
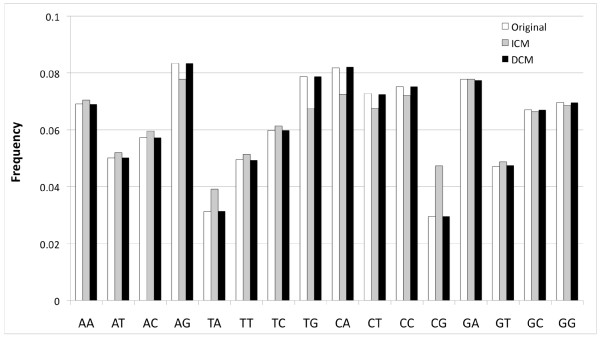
**Comparison of dinucleotide usage under different null models**. The dinucleotide usage of sequences generated by the DCM Markov model (black) and the dinucleotide usage of the original data (white) exhibit Pearson correlation r = 0.9999, in comparison to correlation r = 0.9580 between ICM-generated dinucleotide usage and that of the original sequences. The largest discrepancy is for CpG dinucleotides, for which the ICM-generated frequency is 1.60 times that in the original data. For the DCM-generated sequences, the CpG frequency is 1.0008 times that in the original data.

If each amino acid had only one possible first nucleotide for the underlying codon, then the expected dinucleotide and codon usage in the DCM null model would be exactly equal to those of the reference sequence (see Methods for proof). However, the true genetic code deviates slightly from this behavior (Arginine, Leucine, and Serine can have two possible first nucleotides). To determine how well the DCM preserves dinucleotide and codon usage, we generated a sequence using the DCM Markov model and compared to the properties of the reference sequence. Figure 2 shows the dinucleotide usage of the original sequence and that generated by both the DCM Markov model and the ICM model. The original sequence used was the set of all mouse coding sequences (NCBIM 37; 22,791,336 codons). The DCM and ICM sequences were generated through Python scripts using the Python built-in random number generator. The correlation of the original and ICM dinucleotide frequencies is high (Pearson *r = *0.9580), but the correlation with the DCM value is noticeably superior (Pearson *r = *0.9999). The strongest discrepancy is for CG nucleotides, which are well-known to be hypermutable compared to other dinucleotides. The CG frequency in the ICM is 1.60 times that in the original data, while the DCM CG frequency is only 1.0008 times that in the original data. None of the DCM dinucleotide frequencies differs from its respective original sequence dinucleotide frequency by more than 0.5% of the original sequence value, even though such differences are affected by both systematic biases and finite-size fluctuations.

Preservation of dinucleotide usage inherently implies preservation of codon usage, as shown by the following argument. Define $\tilde{f}\left(\alpha |A\right)$ to be the expected codon usage generated by the Markov process. Then we have:

(1) $$\tilde{f}\left(\alpha |A\right)=\underset{b}{\sum }p\left(\alpha |A,b\right)\tilde{f}\left(b|A\right),$$

where $\tilde{f}\left(b|A\right)$ is the average occurrence of the base *b *3' of amino acid *A *in the sequences generated by the Markov process. Consistent with this, we found that the codon usage was extremely well preserved between the Markov process and the original sequence. There was no codon whose frequency under the DCM differed by more than 0.7% of its value in the original sequence. Because of its accurate accounting for both codon usage and dinucleotide effects, the DCM model was used for all further motif overrepresentation calculations. Since the DCM analysis above indicates that dinucleotide corrections are important we consider the DCM algorithm to be the standard method, and henceforth refer to it by the name CodingMotif.

Would it be better to use a higher order Markov model for the null? 5th order cyclic Markov models are used commonly in gene-finding algorithms, which would suggest they might be appropriate for a null model in motif finding. However, these models were chosen to be 5th order because hexamers were shown to be good for discriminating protein-coding and non-coding regions [27]. This is a criterion unrelated to the present work so such a null model provides no particular advantage for discovering functional motifs. Higher order models also have the drawback of subsuming more of the true signal for motifs into the null, in conflict with the fact that many short motifs are known to be functional. For example, hundreds of 6-mers have been experimentally shown to have a significant effect on exonic splicing activity [28], and microRNA binding sites are only 6-8 bp long [29].

The AA/dinucleotide null has the advantages of being straightforwardly interpretable and of being the lowest order model that accounts for both A A effects and dinucleotide mutation biases. Our emphasis on dinucleotide effects is reasonable because, in many genomes, by far the strongest neutral cause of base-base correlations is the CpG effect, which is known to act on 2 bases at a time [30]. Even in genomes without the CpG effect, there are few 3rd or higher order processes known to be explicitly due to mutation, aside from the special cases of tandem repeats arising from replication slippage or the insertion of transposable elements. Consequently, higher order effects are most reasonably treated as results rather than as part of the null.

### Time scaling

The CodingMotif algorithm takes as input a motif *μ *and a set of independent sequences *S *= {*S*_1_, *S*_2_,...,*S*_*L*_}, corresponding to the coding regions to be analyzed with total sequence length *N*. The algorithm determines and outputs the distribution of the number of occurrences of a motif in sequences that are compatible with the given set of coding regions. The algorithm consists of two parts: first the distribution of each *S*_*i *_is determined, and second these distributions are combined into a single distribution. Each of these parts is analyzed in turn.

Determination of the distribution for each *S*_*i *_is governed by the induction relation 4. Equation 4 calculates a new distribution *D*_*μ*_(*k *+ 1, *X, α*_*k*-Δ+3 _... α_*k*+1_) by adding contributions from at most 6 previously calculated distributions (as there are at most 6 codons compatible with a given amino acid). This calculation is performed for all possible values of *α*_*k*-Δ+3 _... *α*_*k*+1_, yielding at most 6^Δ ^calculations during each stage of the induction. The number of basic operations each induction step requires depends directly on the size of the distribution, which is stored as an array. The size of the distribution is determined by the maximum number of motif occurrences, which is very conservatively bounded by the length of the subsequence, i.e. length(*S*_*i*_). Since len(*S*_*i*_) induction steps are required, an upper bound for the steps required to calculate the distribution of *S*_*i *_is 6^Δ ^length(*S*_*i*_)^2^. We need to do this calculation for all *L *independent sequences, so the total time is also proportional to *L.*

In practice even within a single coding sequence *S*_*i *_we frequently observe sections where no copies of a given motif can possibly occur, due to the structure of the genetic code. These break each sequence *S*_*i *_into much smaller subsequences for which we can calculate the distribution independently, while we can ignore the sections where a motif is forbidden. To see why these subsequences are short, consider a 6-mer motif and its potential occurrence within a stretch of 3 codons. At most, each of these codons has 6-fold degeneracy, so there can be at most 6^3 ^= 216 possible DNA sequences consistent with the given amino acids. If the 6-mer occurs within the three codons, it may overlap in position 1-6, 2-7, 3-8, or 4-9. At most 216 · 4 = 864 motifs may occur within this three codon stretch, while there are 4^6 ^= 4096 possible 6-mer motifs. So at least 79% of 6-mers are forbidden within any three codon stretch. Consequently, regions where a motif is not forbidden will have an approximately geometrically decreasing length distribution. This leads to a much larger number of effective independent regions each with short lengths. We use these effective *S*_*i *_for the distribution function calculations, and this significantly improves the runtime of the algorithm (see Methods: Optimization for sparse motifs). The actual independent regions are a function of the motif, genetic code, and amino acid sequences, and in general there will be *O*(*N*) of them with lengths *O*(1). While it is theoretically possible that some amino acid sequences would necessitate independent regions with longer lengths, such amino acid sequences are exponentially unlikely as long as the amino acid sequences can be approximated as being generated by a finite-length Markov process.

The step of combining the distributions for all independent regions into the overall distribution is rate-limiting. Denote the maximum possible number of motif occurrences in the complete sequence as *n *~ *O*(*N*). Then there can be at most *n *independent regions, and thus at most *n *distributions to be combined. Suppose *n *= 2^*k *^for some *k *(It is irrelevant whether *n *is actually a power of 2 since we can introduce dummy regions without affecting the runtime scaling). Then we can recursively combine the distributions pairwise until only one remains.

The distributions will be combined from smallest to largest size. Consider the worst case scenario in which there are 2^*k *^distributions of size 1. In the first stage we combine these into distributions of size 2. This involves 2^*k*-1 ^pairs of distributions. In the next stage we combine 2^*k*-2 ^pairs of distributions of size 2 into distributions of size 4. Continuing hierarchically, at each stage we combine 2^*k*-*l *^pairs of distributions of size 2^*l*-1 ^for *l *= 1, 2,..., *k *(A more general procedure is to always combine the two smallest distributions, which allows us to handle cases when the distributions vary in size). At a given stage each convolution takes time *O*(2^*l-*1^*log*(*2*^*l-*1^)) using the FFT procedure. The total calculation time is then given by

$$\begin{array}{ll} O\left(\text{running}\;\text{time}\right) & =\overset{k}{\underset{l=1}{\sum }}2^{k-1}O\left(2^{l-1}\text{log}\left(2^{l-1}\right)\right)\;\\ & =2^{k-1}\overset{k}{\underset{l=1}{\sum }}\left(l-1\right)\;\\ & =2^{k-1}\frac{k\left(k-1\right)}{2}\;\\ & =\frac{n}{2}\frac{\text{log}n\left(\text{log}n-1\right)}{2}\;\\ & =O\left(n{\left(\text{log}n\right)}^{2}\right).\;\\ & \; \end{array}$$

So the time requirement for the program is *O*(*n*(log *n*)^2^) = *O*(*N*(log *N*)^2^), much shorter than the exponential number of possible coding sequences.

### Tests of CodingMotif

#### Bacterial motifs

There are two relevant tests for CodingMotif, the first being its ability to more accurately detect over- and under- represented motifs relative to prior methods, and the second being its ability to identify biologically meaningful motifs. For the first type of test, we analyzed the coding sequences of the bacterium *E. coli*. We compared our results to those of Robins et al [19], who used a shuffling-based approach to identify motifs of unusual copy number. Their method involves performing 20-50 shuffles of synonymous codons within each gene to determine the expected copy number of each motif, though their null model does not account for dinucleotide effects. They then identify unusual motifs by comparing the real counts to the shuffled average using the Kullback-Leibler distance, with a z-score threshold based on the standard deviation of counts across shuffled sequences.

Because we used an identical dataset to Robins et al, we were able to directly compare whether our exact approach gives results better than a finite sampling/z-score approach. Robins et al reported a set of 100 over- or under- represented motifs. Among their underrepresented motifs, we found 2 with very weak underrepresentation according to our exact method (underrepresentation p-values CCC: 0.54, CAGAT: 0.31). Moreover, 2 other motifs they call as underrepresented are in fact overrepresented in the data (overrepresentation p-value CTCC: 6e-4, CTGCTGG: 0.075). Among the 31 motifs they report to have unusually high occurrence frequencies, all 31 exhibited very low p-values according to CodingMotif as well(*p *< 0.005), with most exhibiting extremely low p-values (23 motifs between length 3 and 7 with *p *< 10^-8^). However, our exact method detected a total of 251 motifs of lengths between 3 and 7 that have *p *< 10^-8^. These findings indicate that, even with a dataset as large as the coding regions in a bacterial genome, a sampling/z-score approach can have significant error rates, which in this dataset are mostly false negatives. The differences between our exact method and that of Robins et al are somewhat influenced by the lack of dinucleotide effects in the Robins et al null model. When we used an ICM null, which is more similar to the Robins et al null, we found that CodingMotif classifies the motifs CCC, CAGAT, and CTCC similarly as Robins et al. However, under an ICM null, CodingMotif still finds the motif CTGCTGG to be overrepresented (p-value 2e-5), indicating that the misclassification by the Robins et al method is caused by weakness in the sampling/parameterization approach. Moreover, under the ICM null we find a total of 421 motifs of lengths 3-7 with overrepresentation p-values < 10^-8^, demonstrating that the high false negative rate of Robins et al is due to the sampling/parameterization approach rather than the lack of dinucleotide effects in the null.

#### Mammalian splicing motifs

**Figure 3:**
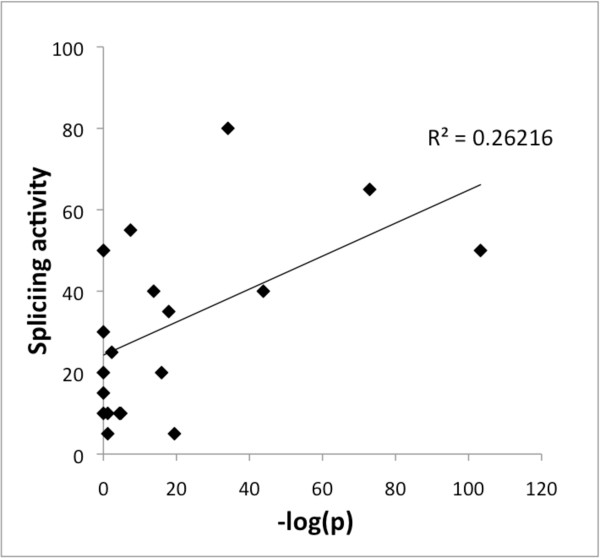
**DCM p-values for motifs with known splicing activity**. We observe a correlation between -log(*p*) for CodingMotif (DCM) p-values and experimentally measured splicing activity (as described in [3]) with *r*^2 ^= 0.26 (t-test p-value 0.02).

As a test of the ability of CodingMotif to identify biologically relevant motifs, we analyzed the behavior of splicing motifs on the coding sequences in human chromosome 1. Our expectation was that motifs with known activity in coding regions, such as exonic splicing enhancers, would show overrepresentation. Figure 3 shows the log p-values from CodingMotif versus experimentally measured exonic splicing enhancer activity, for sequences assayed previously by [3] (activity values rounded to the nearest 5%). The splicing activities refer to rates of splicing rescue when a particular hexamer was inserted into exon2 of a pSXN reporter construct. We found that motifs with superior p-values indeed have greater splicing activities. For example, the motifs with the top 4 p-values all have splicing activities of at least 40%. Overall, we observe a correlation with *R*^2 ^= 0.26 (t-test p-value = 0.02) between - log(*p*) and splicing activity. However, some motifs with strong overrepresentation do not show strong splicing activity. This illustrates the importance of dataset size, as it is likely that this large dataset may have a number of other functional motifs that are overrepresented but have functions unrelated to splicing.

#### Human transcription factor motifs

This issue of dataset size is important for applicability of the method, as a common application for motif detection algorithms is to search for functional motifs in targeted experimental datasets such as determined by chromatin or RNA immunoprecipitation. Because this type of dataset is typically smaller than the genome-scale sets described in the above examples, it can provide a more stringent and practical test of the effectiveness of a motif evaluation program. Neither Itzkovitz et al [17] or Robins et al [19] analyzed such targeted functional sets, instead focusing on whole genome data. We analyzed ChIP-seq data for the human transcription factors GABP and NRSF in the human Jurkat cell line, using data from [31]. For each of these transcription factors, the canonical binding motif is known, as described in [31]. We extracted the sequences around ChIP-seq peaks overlapping coding regions for each of these transcription factors, and then applied CodingMotif to determine if the known motif could be recovered. Since data were from the human genome, we used the full set of coding sequences in the human genome to calculate the null model.

**Figure 4:**
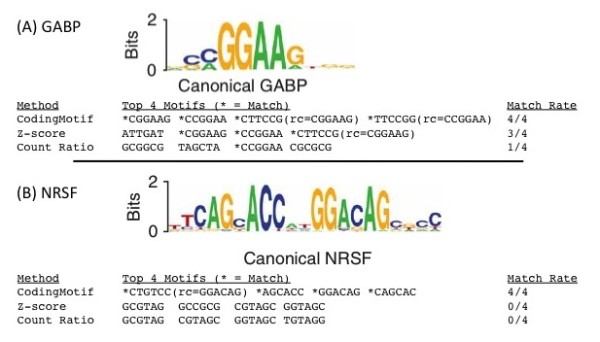
**Comparison of CodingMotif and parametric methods for known binding motifs**. A) All 4 of the top 4 motifs predicted by CodingMotif p-value are exact matches to the canonical motif for the human transcription factor GABP. For comparison, 3 of the top 4 motifs ranked by z-score, and 1 of the top 4 motifs ranked by the ratio of counts in the real sequence to the average in the null distribution, match the GABP canonical motif. B) All 4 of the top 4 motifs predicted by CodingMotif p-value match the canonical motif for NRSF. For motifs ranked by z-score 0/4 of the top motifs match the canonically known motif. 0/4 of the top motifs ranked by count-ratio match the canonically known motif.

Results for GABP are shown in Figure 4A, with the previously known canonical motif shown in weblogo form [31]. The signal for the canonical GABP motif is essentially 7bp long with little degeneracy (CCGGAAG). We determined the top 4 6-mer motifs from CodingMotif as ranked by their overrepresentation p-values, each of which was 1e-21 or better. We found that these 4 motifs were the 4 possible perfect 6-mer matches to the canonical motif: CGGAAG, CCGGAA, and their reverse complements CTTCCG and TTCCGG. To determine whether a parametric approximation of the count distribution would perform equally well, we also calculated z-scores for each 6-mer based on their observed counts and their average counts over all possible coding sequences, which we determined directly from the distribution function calculated by CodingMotif. Note that the mean calculated according to this method is is the ideal of what would be found with an infinite amount of sampling. We found that the top 4 motifs produced by a z-score approach returned only 3/4 of the canonical GABP hexamers. We also sorted motifs according to their count ratio (observed counts/mean counts in the control distribution), the statistic used by Itzkovitz et al [17] to identify motifs. We found that count ratio yielded only 1/4 of the canonical hexamers among the top 4. Thus CodingMotif gives superior results to this idealized parametric method. Methods which parameterize the count distribution from a finite sample set can do no better than this parametric ideal.

We performed a similar test for the transcription factor NRSF also using data from [31], and the results are shown in Figure 4B. NRSF has a bipartite motif, essentially made up of an 8-mer (TCAGCACC) and a 6-mer (GGACAG). The top 4 motifs returned by CodingMotif (each with p-value 1e-13 or better) all matched to 6-mers in these canonical sequences. Two matched to the 8-mer portion (AGCACC and CAGCAC) and two matched to the 6-mer (GGACAG and its reverse complement CTGTCC). However, the z-score measure performed considerably worse. None of the top 4 motifs ranked by z-score matched to the canonical 8-mer or 6-mer. Similarly, none of the top 4 motifs ranked by the count ratio matched to these canonical motifs. These results demonstrate that CodingMotif is superior to parametrically-based methods for protein immunoprecipitation-sized datasets.

We have reported results for 6-mers rather than longer k-mers because we observed that for *k *> 6, a large fraction of k-mers did not appear in the data and many k-mers also had expected copy numbers much less than 1. These properties led to large numbers of outliers in the motif z-score and count ratio statistics. CodingMotif p-values did not suffer from this problem. For example the top GABP 7-mer was the canonical 7-mer CCGGAAG, but the 7-mers with the highest z-scores and count ratios were all low copy number motifs not matching the canonical 7-mer. Although z-scores in principle correct for small copy number effects, these corrections fail when there are strong non-Gaussian deviations in the count distribution. CodingMotif is by construction immune to this problem, and this is likely one of the reasons for its superior results.

We also analyzed whether sampling without resorting to parametric approximations could yield accurate motif predictions. For the GABP dataset, we obtained 100 randomized dicodon shuffles of the data using the method of [23]. We then calculated the fraction of shuffles showing at least as many copies of the motif as found in the original sequence (the non-parametric p-value). The 4 canonical 6-mers had better than average p-values according to this approach (CGGAAG = 0.02; CTTCCG = 0.02; CCGGAA = 0, TTCCGG = 0.09), but in fact 1643 of the possible 4096 hexamers had p-values of 0.02 or better. Thus the only motif which could be distinguished in this approach was CCGGAA, and with a confidence only proportional to the number of shuffles. The many k-mers with low p-values are likely due to a large fraction of the bases in the sequence being under selective pressure in this dataset, a behavior which would distort the null model associated with codon-shuffling. Similar results were found for the NRSF data. Of the 4 motifs found by CodingMotif, the one with the best p-value in this shuffling approach was CTGTCC (*p *= 0.02). However again a very large number of the 6-mers (1908/4096) showed p-values of 0.02 or better. These findings demonstrate the superiority of an externally defined null model as implemented in CodingMotif over one based on shuffling, even in the absence of parameterization.

#### Evaluation on synthetic data

Finally, we tested CodingMotif on synthetic data to estimate what types of counts may be necessary for it to successfully identify motifs. We generated 20 random sequences each 350 codons long (comparable to real protein lengths) according to the DCM Markov model using human coding sequences to train the null and assuming that the 3'-most codon was a stop codon. We then picked from 1-15 of these sequences and inserted a copy of the motif into each by replacing randomly chosen positions. If replacement would create a premature stop codon, another location was chosen. We performed this test for each of the 4096 6-mers 10 times for each number of inserted motifs ranging from 1 to 15. The average and standard deviation of log *p *were calculated across 6-mers and trials. CodingMotif calculations took 21 minutes on a 2.8 GHz dual core Poweredge system without making use of multithreading.

**Figure 5:**
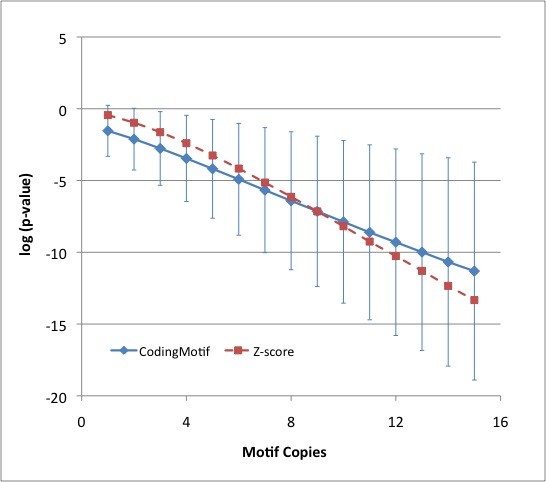
**Motif p-values on synthetic data**. For a randomly generated set of 20 sequences of 350 codons, copies of a motif were overwritten onto random positions within the sequences. CodingMotif and ideal z-score based p-values as a function of the number of inserted copies were calculated. This procedure was performed 10 times for each of the 4096 possible 6-mers. CodingMotif plotted values indicate average and standard deviation of log p-values. Z-score plotted values indicate the value of the erfc function when applied to the average z-score. Standard deviations of z-score based p-values were similar to those of CodingMotif (data not shown).

For 6-mers, a p-value better than 4^-6 ^= 0.0002 is an appropriate significance threshold taking into account multiple testing. As can be seen in Figure 5, this level of significance is achieved, on average, when 6-7 of the sequences have a copy of the motif, though there is strong variability from motif-to-motif as indicated by the plotted standard deviations. For larger datasets, a relatively lower motif density would be expected to be sufficient for detection of significant overrepresentation.

For comparison, we also calculated the average z-score for each motif across these runs, where the z-score was calculated from the exactly enumerated distribution returned by CodingMotif. We observed that the z-score based p-values were systematically too weak (by about one order of magnitude) at 9 or fewer inserted motif copies, though as for CodingMotif there was strong variation from motif-to-motif (data not shown). While CodingMotif tends to exhibit greater sensitivity at these lower copy numbers, this systematic effect is probably less important than the fact that CodingMotif p-values are more accurate for individual motifs. For greater than 9 inserted copies, z-score based p-values are systematically lower than those of CodingMotif. However, both CodingMotif and the z-score method have very significant p-values (much less than 4^-6^) at this range of copy numbers, so this systematic difference is again probably less important than the differences for individual motifs.

#### Human synonymous constraint elements

Recently, Lin et al developed a method to detect elements in coding regions likely to be under constraint based on their synonymous conservation across 29 mammalian genomes (SCEs) [32]. We analyzed whether these regions contained overrepresented motifs. Lin et al reported that these regions had relatively weak compositional biases relative to other coding regions, e.g. with only a ~ 3% difference in GC content between SCE regions and control regions, and significant but small enrichment for known functional motifs such as exonic splicing enhancers and miRNA seeds (< 10% enrichment in each of several datasets). However, we found a number of motifs with extremely strong enrichment p-values in SCEs compared to the human genome coding sequence background, including 13 motifs with p-value 1e-30 or better and each having more than 2 times the number of copies expected by chance. Notably, these include several motifs with multiple CpG dinucleotides. The strong enrichment of specific motifs in these datasets indicates the importance of further motif studies in human coding regions. Note that this dataset (458586 bases) was considerably larger than the synthetic datasets but calculating overrepresentation for all 4096 6-mers was feasible on a standard laptop computer (Macbook Pro 2.66 GHz Intel Core 2 Duo, 330 minutes).

### Software usage and caveats

A software implementation of CodingMotif is available at bioinformatics.bc.edu/chuanglab/codingmotif.tar. We have extended the algorithms described above to allow CodingMotif to calculate p-values for degenerate motifs (e.g. AGACT[A/G]) defined by a set of k-mers. These can be evaluated together, such that an occurrence of any of the k-mers constitutes a match to the degenerate motif. This requires only a minor modification to the counting procedure in the calculation of the distribution function. Note that this k-mer set approach is more general than using IUPAC symbols to handle degeneracy, since IUPAC symbols cannot handle base correlations within a motif. The k-mer set functionality can also be used to handle motifs that could appear on either the forward or reverse strand, e.g. by placing reverse complements such as [AACCTG/CAGGTT] together in a set. In addition, we have written a wrapper allowing CodingMotif to evaluate multiple motifs, each of which may be defined by a set of k-mers, in succession. CodingMotif has been written to handle arbitrary-sized motifs, so motifs of any length can be used as input. For a given run, CodingMotif can return the motif count in the input, its p-value, the count distribution in the null, the mean number of counts in the null, and the z-score for the motif. Underrepresentation p-values can be straightforwardly calculated as 1 minus the overrepresentation p-value. We have demonstrated that p-values for all 4096 6-mers can be calculated for dataset sizes on the scale of several hundred kb in a few hours on a single workstation. Calculations for larger datasets can be trivially parallelized using multiple processors by distributing motif runs across CPUs. The code is open source in C++.

CodingMotif takes fasta files as input. Note that input sequences which are not made up of full codons are conceptually inconsistent with the amino acid-conditioned null model, as hanging bases can match with many possible amino acids. The ends of sequences beginning/ending out of the canonical codon frame should be repaired to full codons before input to CodingMotif, e.g. by truncation of hanging ends. Full documentation for CodingMotif can be found in the downloadable tar file.

It is worth discussing what types of motifs CodingMotif will work best for. The results on NRSF and GABP are based on overrepresentation of exact 6-mers, which are appropriate because binding sites for these two transcription factors both have a relatively strong signal for exact 6-mer sequences as evidenced in their sequence logos (Figure 4). For motifs of greater degeneracy or motifs of different length, the results of CodingMotif would be improved by also testing non 6-mers or degenerate motifs using the software features described above. However, allowing for degeneracy and different lengths also leads to stronger p-value requirements to correct for multiple-testing. These issues may be important for some transcription factors, since transcription factor binding sites may be as long as 15 binding sites [33] with varying levels of degeneracy at internal positions. These issues will be less important for motifs likely to have little degeneracy, such as microRNA binding motifs. In general, we expect CodingMotif to have the greatest advantage over sampling/parametrically-based approaches if the expected and observed number of copies of the motif are both relatively small. This is because CodingMotif is generally more sensitive than z-score approaches when there are low numbers of extra motif copies (Figure 5), and also because non-Gaussian deviations distort z-score approaches when the expected number of copies is small.

Similar issues also affect the power of CodingMotif for building a target classifier. For example, a simple type of classification would be whether a sequence does or does not have a copy of a motif determined to be overrepresented by CodingMotif. For the GABP data, we observe that 65% of the sequences have a copy of at least one of the top 4 CodingMotif hexamers from Figure 4 (a 'recall' statistic). 59% of the sequences have a copy of at least one of the top 4 hexamers by z-score. There are 163 copies of the top 4 CodingMotif k-mers in the GABP sequences, while 17.6 are expected under the DCM null, corresponding to a precision of 90%. For the top z-score hexamers, there are 133 copies while 14.0 are expected, also yielding a precision of 90%. However, we observe a stronger difference in the power of CodingMotif and z-scores for NRSF. 66% of the NRSF sequences contain at least one copy of the top 4 CodingMotif hexamers, while only 21% of the sequences contain a copy of a top 4 z-score hexamer. This low recall for NRSF z-scores is slightly compensated by an increase in precision (84% for CodingMotif and 94% for z-scores). Similar behavior is observed if we train motifs on half the sequences and evaluate on the other half. Using this approach and classifying based on the top 4 motifs, we find that GABP has recall and precision of 63% and 91% respectively for CodingMotif, and 57% and 90% for z-scores. For NRSF, we observe recall and precision of 49% and 77% for CodingMotif, but only 4% and 25% for z-scores. This strong difference between z-scores and CodingMotif for NRSF but not GABP is due to the larger number of possible hexamers that can induce NRSF binding. This causes the prevalence of each such hexamer to be lower, increasing the importance of exact evaluation. Although we have used a naive classification approach to illustrate this idea, this principle should also affect more sophisticated target classification approaches. In standard approaches to building a classifier, the scores for individual motifs are used in the process of clustering and merging motifs to form a position-specific weight matrix, e.g. as described in [33].

## Conclusions

CodingMotif provides an exact non-parametric method for calculating overrepresentation p-values of motifs in coding regions, a previously unsolved problem. We have shown that CodingMotif is able to accurately detect functional motifs in a variety of prokaryotic and eukaryotic datasets, and in short times accessible on single workstations. Prior works have been based on sampling, an approach limited by the infeasibility of sampling more than a tiny fraction of the sequence space, and by their use of parametric approximations for the motif count distribution. We have demonstrated that CodingMotif performs better than such methods using representative experimental data, including human transcription factor ChIP-seq data overlapping coding regions.

CodingMotif provides a theoretically and empirically improved approach over prior methods to identify unusually overrepresented motifs in coding regions. We expect it to be useful for the study of a wide variety of functional genomic problems, notably DNA-protein binding, RNA-protein binding, and microRNA-RNA binding.

## Methods

### Independent codon model

We first consider a method to identify overrepresented motifs in a coding sequence conditional on the amino acid sequence, under the assumption that each codon in the sequence is independent. Specifically, we calculate the overrepresentation or underrepresentation of a motif in a set of protein-coding sequences of total length *N.*

We are given an amino acid sequence *A *= *A*_1_, *A*_2_,..., *A*_*n *_and a motif *μ*. There are *O*(*e*^*N*^) different coding nucleotide sequences that translate into the same amino acid sequence *A*. Denote *C*_*a *_as the set of all such nucleotide sequences. Let *X *denote the random variable that gives the number of occurrences of the motif *μ *for any sequence {*α*} *= α*_1_, *α*_2_, ..., α_N _∈ *C*_*A*_.

The ~ *e*^*N *^sequences in *C*_*a *_will not contribute equally to the expectation. This is because even in the absence of selection on motifs, amino acids have preferences for codon usage. The null model for codon usage can be set as the codon usage in a reference set, which we typically choose to be the set of all coding sequences genome-wide. This provides a background probabilistic model to weight the ~ *e*^*N *^coding sequences.

A direct enumeration of all ~ *e*^*N *^sequences is prohibitive. Therefore we have devised a dynamic programming approach to exactly calculate the distribution of *X*. The distribution for *X*, which we refer to as *D*(*X*), is stored as an array of values, and can be calculated by

(2) $$D\left(X\right)=\underset{\left\{\alpha \right\}\in C_{A}}{\sum }p\left(\left\{\alpha \right\}\right)\delta \left(X\left(\left\{\alpha \right\}\right)\right).$$

Here *δ*(*X*({*α*}) is a delta function centered at the value *X*({*α*}). The probability of the sequence {*α*} is given by

(3) $$p\left(\left\{\alpha \right\}\right)=\overset{N}{\underset{i=1}{\prod }}p\left(\alpha_{i}\right),$$

where the individual *p*(*α*_*i*_) values are determined from the reference codon usage table for the corresponding amino acid. Since the weightings are conditional on the amino acid sequence, the *p*(*α*_*i*_) values for the codons in a synonymous group sum to one.

The distribution can be calculated by an inductive approach. One calculates the *D*(*X*_*k+*1_) distribution for the motif occurrences in the subsequence defined by the first *k + *1 codons using the *D*(*X*_*k*_) distribution defined by the motif occurrences in the first *k *codons. By iterating through this procedure, one can efficiently calculate *D*(*X*_*N*_), which is the desired distribution *D*(*X*) for the full *N *codon sequence.

To perform the dynamic programming calculation, at a given iteration *k *one will need to keep track of each distribution function of the type *D*(*X*_*k*_) conditioned on the possible codon strings in the last Δ - 1 codons {*α*_*k*-Δ+2 _... *α*_*k*_}. Δ is the maximum number of codons that a given instance of the motif can overlap, i.e. for motif length *l*, Δ = [(*l *- 1)/3] + 1, where [*x*] indicates the greatest integer less than or equal to *x*. We denote these distributions as *D*(*X*_*k*_*, α*_*k*-Δ+2 _...*α*_*k*_).

We will need these distributions for all possible values of the codons {*α*_*k*-Δ+2 _... *α*_*k*_}. Note that since the maximum number of copies of a motif scales with *N*, each corresponding distribution requires *O*(*N*) memory, and the total memory requirement is *O*(*e*^Δ-1^*N*). These distribution functions are used to calculate the number of copies of the motif that would be added by appending the *k + *1^*st *^codon to the first *k *codons.

The induction step requires a convolution calculation using all of the *D*(*X*_*k*_, {*α*_*k*-Δ+2 _... *α*_*k*_}) functions. In this step, one counts the number of copies of the motif in each possible set of Δ codons consistent with the amino acids in positions *k *- Δ + 2 through *k + *1, which allows one to calculate the next set of distribution functions. This counting step accounts for motifs in all reading frames, in contrast to recent shuffling-based algorithms [17]. Formally, we have the induction relation

(4) $$\begin{array}{c} D\left(X_{k+1},\alpha_{k-\Delta +3}\ldots \alpha_{k+1}\right)=\\ \;\underset{\alpha_{k-\Delta +2}}{\sum }p\left(\alpha_{k-\Delta +2}\right)\times D\left(X_{k}-\theta \left(\alpha_{k-\Delta +2}\ldots \alpha_{k+1}\right),a_{k-\Delta +2}\ldots \alpha_{k}\right) \end{array}$$

where *θ *is a function on the Δ codons from *k *- Δ *+ *2 to *k *+ 1 defined as *θ*(*α*_*k-*Δ*+*2 _*... α*_*k+*1_) ≡ the number of copies of the motif that end in the last codon of {*α*_*k*-Δ+2 _... *α*_*k*+1_}. The sum is over all values of *α*_*k*-Δ+2 _consistent with the amino acid *A*_*k-*Δ*+*2_. When the end of the sequence is reached, the final value of *D*(*X*_*N*_) is calculated from the weighted sum of the *D*(*X*_*N*_*, α*) values that cover the last Δ - 1 codons of the amino acid sequence, i.e.

(5) $$\begin{array}{c} D\left(X_{N}\right)=\\ \;\;\;\underset{\alpha_{N-\Delta +2}\ldots \alpha_{N}}{\sum }p\left(\left\{\alpha_{N-\Delta +2}\ldots \alpha_{N}\right\}\right)\times D\left(X_{N},\left\{\alpha_{N-\Delta +2}\ldots \alpha_{N}\right\}\right). \end{array}$$

The probabilities in equation 5 can be calculated directly as

(6) $$p\left(\left\{\alpha_{N-\Delta +2}\ldots \alpha_{N}\right\}\right)=\overset{N}{\underset{i=N-\Delta +2}{\prod }}p\left(\alpha_{i}\right).$$

Note that all of these calculations can be done in either the 5' to 3' or 3' to 5' direction. In practice, we use the 3' to 5' direction, as this is necessitated by the way in which the Dinucleotide-corrected Codon Model (described below) is implemented.

#### Optimization for sparse motifs

For most amino acid sequences, the possible locations of the motif consistent with the genetic code are sparsely distributed. That is, depending on the motif, there can be large portions of the amino acid sequence where no motif is possible for any consistent choice of codons. Inductively calculating the motif occurrence distribution *D *in these regions is clearly wasteful. To take advantage of sparseness, we split our induction up into independent regions that can contain motifs. As described above we can calculate *D*(*X*_*k*_), the motif occurrence distribution for the subsequence *A*_1_*, A*_2_, ..., *A*_*k*_, where *k *<*N*. However, at each step we also check whether there exists any codon choice {*α*} that would allow a motif instance to start in *α*_1_*α*_2 _... *α*_*k *_and end in *α*_*k*+1_*α*_*k*+2 _... α_*N*_. Such a motif must occur in the subsequence *α*_*k*-Δ_*α*_*k*-Δ+1 _... α_*k *_*..*. α_*k *+ Δ-1_*α*_*k*+Δ_. The calculation of whether such a motif exists is then constant time for a fixed motif length.

If a motif instance is possible, we continue the induction. However, if not, then the distribution on the traversed sequence is independent of the distribution for the rest of the sequence. We therefore store the current motif occurrence distribution, denoted as *D*_*c *_*= D*(*X*_*k*_) and scan forward in the sequence until we find the next codon *j *>*k *such that a motif can occur. Considering this as the beginning of a new truncated sequence, $A_{1}^{\prime }A_{2}^{\prime }\ldots A_{N}^{\prime }=A_{j}A_{j+1}A_{N}$, we calculate a new distribution *D*(*X*_*k'*_). This process is repeated until the end of the amino acid sequence. We can calculate the complete distribution by convolving all such distributions. The advantage of this approach is that any regions for which a motif instance is impossible (positions *k + *1 through *j *- 1 in the description above) do not require convolution calculations. For longer motifs, the set of possible motif locations is increasingly sparse, which reduces the calculation time. This time reduction tends to offset the larger number of terms to be calculated in equation 4 for longer motifs.

#### Convolution calculation

A convolution of two distributions can be calculated by considering the values in each distribution as coefficients of two generating functions and then multiplying the two generating functions. Term-by-term multiplication of the two generating functions will take time *O*(*mn*) when the polynomials are of degree *m *and *n. *An alternative approach is a Fast Fourier Transform (FFT), which takes advantage of the fact that convolution in x-space is equivalent to multiplication in Fourier space. In the FFT approach, each polynomial is converted to Fourier space, the transformed polynomials are multiplied, and the product is converted back to x-space [34]. The algorithm takes *O*(*m *log *m *+ *n *log *n*) time.

We tested both the direct and FFT approaches. For the motif lengths we investigated (4 - 7 bp), the FFT approach is not noticeably faster than the direct polynomial multiplication. This is because the convolution calculations involve a large number of multiplications in which *m *≫ *n *and for which log *m *may be comparable to *n. *The FFT approach also occasionally yielded slightly negative values for polynomial coefficients due to limits on computer precision in the transform step. Therefore in the final program we used the direct multiplication approach.

### Dinucleotide-corrected codon model

Because we were concerned that the Independent Codon Model (ICM) did not sufficiently account for neutral dinucleotide biases, we implemented a dinucleotide-corrected codon model (DCM) which includes dinucleotide biases in the null model. The DCM uses a Markov model to generate the sequence, starting from the 3'-most codon and working backward to the 5' end. This choice of direction simplifies the calculation, since for most amino acids specification of the amino acid fixes the 5'-most nucleotide of a codon. Note that although the program is run in this direction, the results sections describe motifs in the standard 5' to 3' direction.

To specify the Markov model, we use the conditional codon usage table as observed in the reference sequence. The probability of choosing a codon is conditioned upon the amino acid of the current codon as well as the first nucleotide of the adjacent 3' codon. Formally, let *F*(*α, b*) be the number of instances in the reference sequence for which one observes the codon *α *followed by base *b*, such that the amino acid coincident with *α *is *A. *Let *F*(*A, b*) be the number of instances for which one observes amino acid *A *followed by base *b *in the reference sequence. Then define

(7) $$p\left(\alpha |A,b\right)=\frac{F\left(\alpha ,b\right)}{F\left(A,b\right)}.$$

In the DCM, sequences are then generated from the 3' to the 5' end with probabilistic weighting

(8) $$p\left(\alpha_{i+1}\right)=\underset{\alpha_{i}}{\sum }p\left(\alpha_{i}\right)*p\left(\alpha_{i+1}|A_{i+1},b\left(\alpha_{i}\right)\right).$$

Here we have written *b*(*α*_*i*_) to refer to the 1st base of codon *α*_*i*_, treating *b *as a function on a codon.

By iterating through equation 8 we can calculate the probability of the complete sequence given the amino acids. One source of ambiguity is how to treat the 3'-most codon. Our rule is to use the first nucleotide 3' to the sequence as the starting point of the probability assignment. This is a minor assumption since for most amino acids the first base of the codon is forced. If the sequence is a whole gene then we require *b *for the 3'-most codon to be "T", which is the first letter of all three stop codons. To avoid arbitrariness in the choice of stop codon, we by convention do not look for motifs overlapping the stop codon. Also, when applying the optimization for sparse motifs, we do not need to use the *p*(*α*) values of codons in the regions where a motif is not possible. Therefore, we use a shortcut that frees us from having to apply equation 8 in such regions. This shortcut is to traceback the *p*(*α*) from the region where a motif is possible to an amino acid where there is only one possible first base of the codon. This typically requires consideration of only a few codons in the 3' direction since for the large majority of amino acids (17/20), specification of an amino acid also specifies the first codon base.

With this approach in mind, calculating the motif occurrence distribution is analogous to the ICM case. One can apply equation 4 but with the substitution of the conditional probability

(9) $$p\left(\alpha_{k-\Delta +2}|A_{k-\Delta +2},b\left(\alpha_{k-\Delta +1}\right)\right)$$

for the probability factor. When the 5' end of the sequence is reached, the final value of *D*_*μ*_(*N, X*) can be calculated using 5, though the calculation of *p*({*α*_*N*-Δ+2 _... *α*_*N*_}) for equation 6 should again use conditional probabilities. For the DCM, this means replacing equation 6 with

(10) $$p\left(\left\{\alpha_{N-\Delta +2}\ldots \alpha_{N}\right\}\right)=p\left(\alpha_{N-\Delta +2}\right)\overset{N}{\underset{i=N-\Delta +3}{\prod }}p\left(\alpha_{i}|A_{i},b\left(\alpha_{i-1}\right)\right).$$

#### Preservation of codon and dinucleotide usage by DCM

The purpose of using the DCM null instead of the ICM was to preserve the dinucleotide frequency and codon usage found in the reference sequence. Here we provide an argument for why the DCM model can closely preserve these quantities. Due to the structure of the genetic code, specification of an amino acid usually fixes the first nucleotide of the underlying codon. Only for the amino acids Ser, Arg and Leu is there degeneracy in the first nucleotide. Here we show that under the simplifying assumption that specifying the amino acid fixes the first nucleotide of the codon for every amino acid, then the codon and dinucleotide usage generated by the Markov process equals the codon and dinucleotide usage in the reference sequence. Because this assumption is approximately true for the real genetic code, codon and dinucleotide usage will be well-preserved by the DCM model.

Suppose in our amino acid sequence that amino acids *A *and *B *occur at locations *i + *1 and *i *respectively. Since by assumption specification of *B *also specifies the first base *b *of the underlying codon, the probability of selecting codon *α *at position *i + *1 is given by

$$\begin{array}{ll} p\left(\alpha |AB\right) & =p\left(\alpha |A,b\right)\;\\ & =\frac{F\left(\alpha ,b\right)}{F\left(A,b\right)}\;\\ & \; \end{array}$$

where the counts *F*(*α, b*) and *F*(*A, b*) are from the reference sequence as defined above.

Denote the number of occurrences of *α *in the original sequence as *F*(*α*), and denote the expected number of occurrences of a codon α in a sequence generated by our Markov process as *E*(*α*). From our definitions it is clear that *F*(*α*) = Σ_*b *_*F*(*αb*). Meanwhile for the Markov process we have

$$\begin{array}{ll} E\left[\alpha \right] & =\underset{B}{\sum }P\left(\alpha |AB\right)F\left(A,B\right)=\underset{b}{\sum }P\left(\alpha |Ab\right)F\left(A,b\right)\;\\ & =\underset{b}{\sum }\frac{F\left(\alpha ,b\right)}{F\left(A,b\right)}F\left(A,b\right)=\underset{b}{\sum }F\left(\alpha ,b\right)\;\\ & =F\left(\alpha \right).\;\\ & \; \end{array}$$

In the second step we have made use of the fact that each amino acid *B *specifies a first base *b *in the codon. Thus we see that the expected number of instances of any codon *α *is equal to the number of copies in the original sequence.

To see that the Markov process preserves dinucleotide counts, we again assume the idealized case in which specification of an amino acid also specifies the first base of the underlying codon. Denote *ψ*_*xy*_(*αb*) as the number of times a dinucleotide *xy *appears within the 4 bases consisting of *α *and the nucleotide *b *corresponding to the first base of the codon underlying amino acid *B. *Thus *ψ *will have a value of 0,1, 2, or 3. Then the total number of copies of the dinucleotide *xy *in the original sequence is

(11) $$F\left(xy\right)=\underset{A,b,\;\;\alpha \;\text{at}\;A}{\sum }F\left(\alpha b\right)\psi_{xy}\left(\alpha b\right).$$

The expected number of copies of *xy *generated by the Markov process *E*[*xy*] can be broken down into contributions *E*[*xy*|*Ab*] corresponding to cases where an amino acid *A *occurs 5' to a base b, i.e.

$$\begin{array}{ll} E\left[xy\right] & =\underset{Ab}{\sum }F\left(A,b\right)E\left[xy|A,b\right]\;\\ & =\underset{Ab}{\sum }F\left(A,b\right)\underset{\alpha }{\sum }P\left(\alpha |A,b\right)\psi_{xy}\left(\alpha b\right).\;\\ & \; \end{array}$$

Plugging in *P*(α|*Ab*) as before, we get

$$\begin{array}{ll} E\left[xy\right] & =\underset{A,b,\alpha }{\sum }F\left(A,b\right)\frac{F\left(\alpha b\right)}{F\left(A,b\right)}\psi_{xy}\left(\alpha b\right)=\underset{A,b,\alpha }{\sum }F\left(\alpha b\right)\psi_{xy}\left(\alpha b\right)\;\\ & =F\left(xy\right)\;\\ & \; \end{array}$$

### Coding and UTR lengths

For the initial coding and UTR length analysis, all gene transcripts from the human genome were downloaded from Ensembl v63. Lengths were calculated using all transcripts having simultaneous 5' UTR, 3' UTR, and coding region annotations. The observed lengths were: 5' UTR 180 bp (σ = 340 bp), 3' UTR 820 bp (*σ *= 1020 bp), coding region 960 bp (*σ *= 750 bp).

### GABP and NRSF analysis

We downloaded ChIP-seq peaks for the NRSF monoclonal antibody and GABP datasets of [31]. We defined ChIP-seq regions as the 50 bp upstream and downstream of the annotated peak. We intersected these 100 bp regions with the set of UCSC known human coding sequences in human build Hg18. Unique regions with an overlap of at least 50bp with a coding sequence were retained. Codons overlapping these regions were obtained using the "Extract Features" tool in GALAXY [35]. These codon annotations were used to truncate the ends of the regions by retaining portions of the sequence overlapping a complete codon. Regions with ambiguous coding frames were removed from the dataset. Most calculations were performed in GALAXY with additional minor processing using PERL scripts. For the count ratio statistic, we considered only motifs with at least 3 copies in the datasets to reduce bias from outliers. None of these motifs with fewer copies were matches to the canonical motifs. The input data for CodingMotif corresponded to a very small subset of the original ChIP-seq data, as most of the original ChIP-seq peaks were in noncoding regions. For GABP the input data to CodingMotif was 134 sequences (from 6442 original ChIP-seq peaks). For NRSF the input data to CodingMotif was 101 sequences (from 2596 original ChIP-seq peaks).

### Human synonymous constraint elements

The SCE9 dataset was obtained from [32]. Codons overlapping these sequences were obtained using GALAXY and regions with ambiguous coding frames were removed before being used as input for CodingMotif.

## Authors' contributions

YD contributed to the design of the algorithms, wrote software, and contributed to the writing of the manuscript. WL contributed to the design of the algorithms, wrote software, and contributed to the writing of the manuscript. JHC contributed to the design of the algorithms, contributed to the writing of the manuscript, and oversaw the project. All authors read and approved the final manuscript.
